# Hypoglycemic and beta cell protective effects of andrographolide analogue for diabetes treatment

**DOI:** 10.1186/1479-5876-7-62

**Published:** 2009-07-16

**Authors:** Zaijun Zhang, Jie Jiang, Pei Yu, Xiangping Zeng, James W Larrick, Yuqiang Wang

**Affiliations:** 1 Institute of New Drug Research, Jinan University College of Pharmacy, Guangzhou, 510632, PR China; 2 Panorama Research Institute, 1230 Bordeaux Drive, Sunnyvale, CA 94089, USA

## Abstract

**Background:**

While all anti-diabetic agents can decrease blood glucose level directly or indirectly, few are able to protect and preserve both pancreatic beta cell mass and their insulin-secreting functions. Thus, there is an urgent need to find an agent or combination of agents that can lower blood glucose and preserve pancreatic beta cells at the same time. Herein, we report a dual-functional andrographolide-lipoic acid conjugate (AL-1). The anti-diabetic and beta cell protective activities of this novel andrographolide-lipoic acid conjugate were investigated.

**Methods:**

In alloxan-treated mice (a model of type 1 diabetes), drugs were administered orally once daily for 6 days post-alloxan treatment. Fasting blood glucose and serum insulin were determined. Pathologic and immunohistochemical analysis of pancreatic islets were performed. Translocation of glucose transporter subtype 4 in soleus muscle was detected by western blot. In RIN-m cells *in vitro*, the effect of AL-1 on H_2_O_2_-induced damage and reactive oxidative species production stimulated by high glucose and glibenclamide were measured. Inhibition of nuclear factor kappa B (NF-κB) activation induced by IL-1β and IFN-γ was investigated.

**Results:**

In alloxan-induced diabetic mouse model, AL-1 lowered blood glucose, increased insulin and prevented loss of beta cells and their dysfunction, stimulated glucose transport protein subtype 4 (GLUT4) membrane translocation in soleus muscles. Pretreatment of RIN-m cells with AL-1 prevented H_2_O_2_-induced cellular damage, quenched glucose and glibenclamide-stimulated reactive oxidative species production, and inhibited cytokine-stimulated NF-κB activation.

**Conclusion:**

We have demonstrated that AL-1 had both hypoglycemic and beta cell protective effects which translated into antioxidant and NF-κB inhibitory activity. AL-1 is a potential new anti-diabetic agent.

## Introduction

Diabetes mellitus has become an epidemic in the past several decades owing to the advancing age of the population, a substantially increased prevalence of obesity, and reduced physical activity. The US Center for Disease Control and Prevention (CDC) estimates that 20.8 million children and adults (7.0% of the US population) had diabetes in 2005 http://www.cdc.gov/diabetes/pubs/general.htm. Of this total, 1.5 million were newly diagnosed and over 30% (6.2 million) were undiagnosed. In addition, 54 million people are estimated to have pre-diabetes. Among those diagnosed with diabetes, 85% to 90% have type 2 diabetes.

Type 1 diabetes is characterized by insulin deficiency, a loss of the insulin-producing beta cells of the pancreatic islets of Langerhans. Beta cell loss is largely caused by a T-cell mediated autoimmune attack [1]. Type 2 diabetes is preceded by insulin resistance or reduced insulin sensitivity, combined with reduced insulin secretion. Insulin resistance forces pancreatic beta cells to produce more insulin, which ultimately results in exhaustion of insulin production secondary to deterioration of beta cell functions. By the time diabetes is diagnosed, over 50% of beta cell function is lost [2]. The gradual loss of beta cell function results in increased levels of blood glucose and ultimate diabetes.

Recent availability of expanded treatment options for both types 1 and 2 diabetes has not translated into easier and significantly better glycemic and metabolic management. Patients with type 1 diabetes continue to experience increased risk of hypoglycemic episodes and progressive weight gain resulting from intensive insulin treatment, despite the availability of a variety of insulin analogs. Given the progressive nature of the disease, most patients with type 2 diabetes inevitably proceed from oral agent monotherapy to combination therapy and, ultimately require exogenous insulin replacement. Both type 1 and type 2 diabetic patients continue to suffer from marked postprandial hyperglycemia. None of the currently used medications reverse ongoing failure of beta cell function [3]. Thus, there is an urgent need to find an agent/combination of agents that can both lower blood glucose and preserve the function of pancreatic beta cells.

*Andrographis paniculata *(*A. paniculata*) is a traditional Chinese medicine used in many Asian countries for the treatment of colds, fever, laryngitis and diarrhea. Studies of plant extracts demonstrate immunological, antibacterial, antiviral, anti-inflammatory, antithrombotic and hepatoprotective properties [4-8]. In Malaysia, this plant is used in folk medicine to treat diabetes and hypertension [9]. An aqueous extract of *A. paniculata *was reported to improve glucose tolerance in rabbits, and an ethanolic extract demonstrated anti-diabetic properties in streptozotocin (STZ)-induced diabetic rats [10].

**Figure 1:**
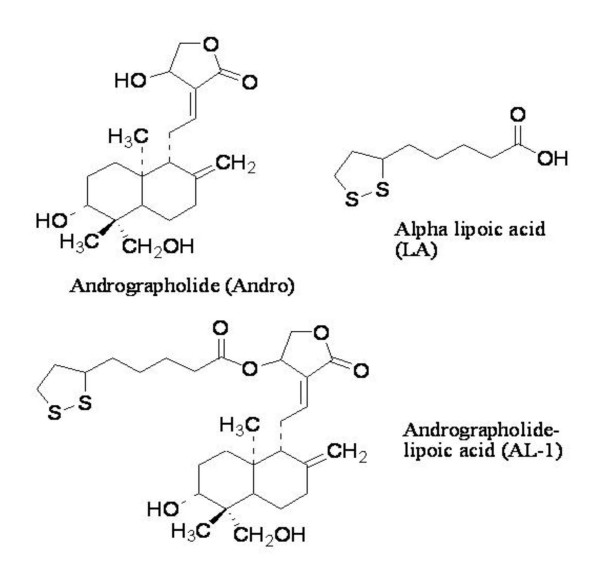
**Structures of Andro, LA and AL-1**.

Androdrographolide (Andro, Fig. 1), the primary active component of *A. paniculata*, lowers plasma glucose in STZ-diabetic rats by increasing glucose utilization [11]. The db/db diabetic mice progressively develop insulinopenia with age, a feature commonly observed in late stages of human type 2 diabetes when blood glucose levels are not sufficiently controlled [12]. When an Andro analog was administered orally to db/db mice at a dose of 100 mg/kg daily for 6 days, the blood glucose level decreased by 64%, and plasma triglyceride level by 54% [13]. These data showed that *A. paniculata *and Andro had significant activity for diabetes.

Alpha-lipoic acid (LA, 1, 2-dithiolane-3-pentanoic acid, Fig. 1), is one of the most potent antioxidants. Pharmacologically, LA improves glycemic control and polyneuropathies associated with diabetes mellitus, as well as effectively mitigating toxicities associated with heavy metal poisoning [14,15]. As an antioxidant, LA directly terminates free radicals, chelates transition metal ions (e.g., iron and copper), increases cytosolic glutathione and vitamin C levels, and prevents toxicities associated with their loss. These diverse actions suggest that LA acts by multiple mechanisms both physiologically and pharmacologically. For these reasons, LA is one of the most widely used health supplements and has been licensed and used for the treatment of symptomatic diabetic neuropathy in Germany for more than 20 years.

Realizing the beneficial mechanisms of action and effects of both Andro and LA for treatment of diabetes, we conducted experiments to evaluate the efficacy and possible mechanism(s) of action of a conjugate of Andro and LA, i.e., andrographolide-lipoic acid conjugate (AL-1, Fig. 1), *in vitro *and in experimental diabetic animal models.

## Methods

### Reagents

AL-1 was synthesized and purified in our laboratory [16]. Andro, LA, DMSO and glibenclamide were purchased from Alfa Aesar (War Hill, MA, USA). Alloxan, leupeptin, luminol were purchased from Sigma-Aldrich Corp. (St Louis, MO, USA). pNF-κB-luc, PRL-TK plasmid and dual luciferase reporter (DLR) assay kits were purchase from Promega Corp. (Madison, WI, USA). Lipofectamine 2000 and Opti-MEM medium were purchased from Invitrogen Corp. (Carlsbad, CA, USA). Mouse IL-1β and IFN-γ were purchased from PeproTech (Rocky Hill, NJ, USA). Polyclone anti-GLUT4 antibody was purchased from Chemicon International Inc. (Temecula, CA, USA). Polyclone anti-insulin antibody, ployclone anti-β-actin antibody and HRP-conjugated goat anti-rabbit antibody were purchased from Beijing Biosynthesis Biotechnology Co. Ltd. (Beijing, China).

### Diabetic mouse model

Female BALB/c mice, aged 6–8 weeks (18–22 g), were obtained from the Experimental Animal Center of Guangdong Province, China (SPF grade). Mice were housed in an animal room with 12 h light and 12 h dark, and were maintained on standard pelleted diet with water *ad libitum*. After fasting for 18 h, mice were injected via the tail vein with a single dose of 60 mg/kg alloxan (Sigma-Aldrich), freshly dissolved in 0.9% saline. Diabetes in mice was identified by polydipsia, polyuria and by measuring fasting serum glucose levels 72 h after injection of alloxan. Mice with a blood glucose level above 16.7 mM were used for experiments.

Diabetic mice were randomly divided into 6 groups of 6 mice. The first group was given vehicle (20% DMSO in distilled water) as a diabetic control group; the 2nd, 3rd and 4th groups were given AL-1 at doses of 20, 40 and 80 mg/kg, respectively; the 5th group was given Andro at 50 mg/kg (equal molar dose to 80 mg/kg AL-1); the 6th group was given glibenclamide at 1.2 mg/kg as a positive control. And 6 non-diabetic mice received vehicle as a normal control group. On the 4th day after alloxan administration, fasting (12–14 h) blood glucose levels were measured using a complete blood glucose monitoring system (Model: SureStep, LifeScan, Johson-Johson Co., Shanghai, China). AL-1, Andro, glibenclamide and vehicle were given by intragastric administration once daily for 6 days, respectively. On the evening of day 6, all mice were fasted overnight (12–14 h), and the following morning, after blood glucose of all groups was measured, animals were killed by decapitation. Blood was collected by drainage from the retroorbital venous plexus and kept on ice. Pancreas and soleus muscle were removed and immediately frozen at -80°C for various assays. Clotted blood samples were centrifuged at 3,000 × g for 15 min to obtain serum. The levels of serum insulin were determined by chemiluminescent immunoassay using a commercially available kit (Beijing Atom HighTech Co., Ltd., Beijing, China).

### Pathologic and immunohistochemical analysis of pancreas

Pancreatic tissues were collected and placed in fixative (40 g/l formaldehyde solution in 0.1 M PBS) overnight, and was washed with 0.1 M PBS, then paraffin embedded, sectioned (2 μm), and stained with hematoxylin and eosin. For immunostaining studies, rabbit anti-mouse insulin antibody (1:50; Beijing Biosynthesis Biotechnology Co. Ltd.) was incubated with the sample sections for 3 h at 37°C. Horseradish peroxidase (HRP)-conjugated goat anti-rabbit IgG antibody (1:200; Beijing Biosynthesis Biotechnology Co. Ltd.) was used for 3, 3'-diaminobenzidine (DAB) coloration. Area of pancreatic islet was analyzed using Olypus analySIS image analysis software (Olympus Optical Co., Tokyo, Japan).

### Western blot analysis of glucose transporter subtype 4 (GLUT4) translocation

GLUT4 protein extract was prepared as described in Takeuchi et al. [17] with modifications. Briefly, soleus muscles were homogenized in an ice-cold buffer containing 20 mM HEPES, 250 mM sucrose, 2 mM EGTA, 0.2 mM phenylmethylsulfonyl fluoride (PMSF), and 1 μM leupeptin (Sigma-Aldrich) at pH 7.4. Nuclei and unbroken cells were removed by centrifugation at 2,000 × g for 10 min. Total membrane fraction was prepared by centrifugation of the supernatant in a super-speed centrifuge at 190,000 × g for 1 h at 4°C. The membrane pellets were re-suspended in homogenization buffer and stored at -80°C. Immunoblotting was performed using polyclonal anti-GLUT4 antibody (1:2,000 dilution; Chemicon) at 4°C overnight, and polyclonal anti-actin antibody (1:500 dilution; Beijing Biosynthesis Biotechnology Co. Ltd.) was used as an inter-control. After washing with TBS-T, the blots were incubated for 1 h at room temperature with HRP-conjugated goat anti-rabbit antibodies (1:2,000 dilution; Beijing Biosynthesis Biotechnology Co. Ltd.), and were detected using ECL Plus (PIERCE, Rockford, IL, USA).

### Cell culture

RIN-m cell is an insulinoma cell line derived from a rat islet cell tumor [18]. Cells were purchased from the American Type Culture Collection and grown at 37°C in a humidified 5% CO_2 _atmosphere in DMEM (Gibco/BRL, Grand Island, NY, USA) supplemented with 10% fetal bovine serum, 2 mM glutamine, 100 units/ml of penicillin, and 100 μg/ml of streptomycin.

### Cell viability by MTT assay

RIN-m (5 × 10^4 ^cells/ml, 100 μl/well) were plated in 96-well plates. After incubation for 24 h, cells were pretreated with Andro, LA and AL-1 for 1 h. An equal volume of 1% DMSO was added as a vehicle control (DMSO final concentration to 0.1%). Then, 500 μM H_2_O_2 _were added, and the cells were incubated for another 24 h to induce cell injury. Viability of cultured cells was determined by MTT assay.

### ROS inhibition assay

Luminol chemiluminescence (CL) was used to evaluate intracellular oxidant production. RIN-m cells were planted in 96-well plates and cultured in DMEM containing 10% fetal bovine serum and 450 mg/dl glucose. When cells reached the loose confluent layer, medium was replaced with DMEM containing 1% FBS and 100 mg/dl glucose for 24 h. The cells were then exposed to 100, 275 and 450 mg/dl glucose or 0.1, 1 and 10 μM glibenclamide under the presence of 100 mg/dl glucose for 2 h or pretreated with Andro, LA and AL-1 at a concentration of 1 μM for 1 h and exposed to 450 mg/dl glucose or 1 μM glibenclamide for another 2 h. After treatment, 1 mM luminol (in DMSO) was added to the cells (final concentration of 50 μM). The time luminol was added was recorded as time "0", and relative luminescence units (RLU) were measured for 10 s every 2 min for a total of 30 min on a luminometer (TECAN, Männedorf, Switzerland). The areas under the chemiluminescence curves (AUC_CL_) measured from time "0" to 30 min after adding luminol were calculated using an Orange software (OriginLab, Jersey, NJ, USA).

### NF-κB assay by DLR system

RIN-m cells (1 × 10^5 ^cells/ml, 400 μl/well) in growth medium (high glucose DMEM containing 10% FBS) were plated in a 24-well plate, and were incubated for 24 h. Plasmid pNF-κB-luc and PRL-TK (Promega) in a ratio of 50:1 were co-transfected into RIN-m cells as described by the transfection guideline of lipofectamine 2000 (Invitrogen), and cultured in Opti-MEM medium (Invitrogen) for 4 h. Then medium was changed with the growth medium, and the cells were cultured for another 12 h. Andro, LA, AL-1 or vehicle control (DMSO final concentration to 0.1%) was added (final concentration: 1 μM) to pre-treat cells for 1 h. IL-1β (5 ng/ml, PeproTech) and IFN-γ (50 ng/ml, PeproTech) were then added, and the cells were incubated for another 24 h. NF-κB expression was determined by the dual luciferase reporter (DLR) assay kits (Promega).

### Statistics

Data were expressed as the mean ± S.D. for the number (n) of animals in the group as indicated in table and figures. Repeated measures of analysis of variance were used to analyze the changes in blood glucose and other parameters. Compare value less than 0.05 was considered significant.

## Results

### AL-1 attenuates alloxan-induced diabetes

Alloxan specifically targets pancreatic beta cells, where it induces ROS, destroying the beta cells to cause diabetes. Mice administered 60 mg/kg, i.v. of alloxan became hyperglycemic after 3 days. The blood glucose reached 27.0 ± 1.2 mM (Table 1), a value within the acceptable diabetic range. Drugs were administered, i.g. starting on day 3 and continued daily for 6 days. On day 7, mice were sacrificed, and various assays were performed.

**Table 1:** Effect of AL-1 on blood glucose level in alloxan-induced diabetic mice.

Groups	Blood glucose level (mM)		
	
	Day 0	Day 6	Changes (%)
Normal control	5.8 ± 1.5	5.9 ± 1.7	+1.7
Diabetic control	27.0 ± 1.2 ^a^	25.4 ± 7.8	-5.9
Diabetic + AL-1 (20 mg/kg)	24.9 ± 3.1^a^	16.8 ± 2.4 ^b^	-32.5
Diabetic +AL-1 (40 mg/kg)	25.0 ± 2.7 ^a^	13.9 ± 3.4 ^c^	-44.4
Diabetic + AL-1 (80 mg/kg)	24.6 ± 3.2 ^a^	8.6 ± 3.1 ^c, d^	-65.0
Diabetic + Andro (50 mg/kg)	24.8 ± 3.0 ^a^	16.8 ± 2.1 ^b^	-32.3
Diabetic + Gli (1.2 mg/kg)	24.7 ± 5.1 ^a^	10.1 ± 3.0 ^c, d^	-59.1

72 h after alloxan administration (Day 0), drugs were given by intragastric administration once daily for 6 days. On day 0 and day 6, fasting blood glucose levels were determined. Values are means ± S.D. of 6 mice. ^a^*P *< 0.01 *vs*. normal mice; ^b^*P *< 0.05 *vs*. value on day 0; ^c^*P *< 0.01 *vs*. value on day 0; ^d^*P *< 0.05 *vs*. Andro treatment on day 6. Gli: glibenclamide.

#### AL-1 significantly lowers blood glucose

AL-1 markedly decreased blood glucose levels in diabetic mice in a dose-dependent manner (Table 1). At 20, 40, and 80 mg/kg, AL-1 decreased blood glucose by 32.5, 44.4, and 65.0%, respectively. This hypoglycemic effect was equal to that of glibenclamide, a widely used anti-diabetic agent. AL-1 was 2-fold more potent than its parent compound Andro. For example, at an equal molar dose, AL-1 (80 mg/kg) lowered blood glucose by 65% while its parent Andro (50 mg/kg) only lowered blood glucose by 32.3%.

#### AL-1 augments insulin levels

**Figure 2:**
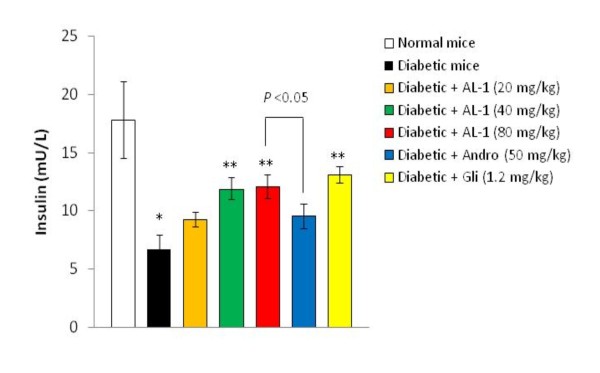
**Effect of AL-1 on serum insulin level in diabetic mice**. Alloxan-induced diabetic mice were treated with AL-1, Andro or glibenclamide by intragastric administration once daily for 6 days. On day 6, serum insulin levels were detected. Each column represents the mean ± S.D. of 6 mice. *P < 0.05 *vs*. normal group, **P < 0.01 *vs*. diabetic group. Gli: glibenclamide.

The diabetic animals had a significantly reduced level of insulin (Fig. 2). Administration of AL-1 dose-dependently increased insulin levels. Glibenclamide had a similar activity in diabetic mice and normal ones. Andro had a modest effect that did not reach statistical significance.

#### AL-1 preserves pancreatic beta cell morphology and function

**Figure 3:**
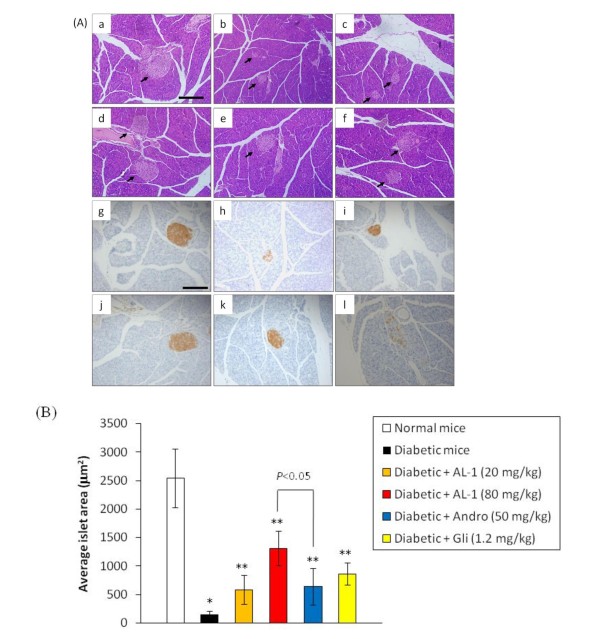
**Pathologic and immunohistochemical analysis of mouse pancreas**. Alloxan-induced diabetic mice were treated with Andro, AL-1 or glibenclamide for 6 days, the the pancreas were isolated for hematoxylin and eosin staining or anti-insulin immuohistaining. A, Representative morphology of pancreatic islets. a-f: hematoxylin and eosin staining. Arrow showed the islets' position, scale bar: 50 μm; g-l: immunostaining against insulin as visualized by the HRP-DAB method, scale bar: 50 μm. a, g, no-diabetic control; b, h, diabetic + vehicle control; c, i, diabetic + AL-1 20 mg treatment; d, j, diabetic +AL-1 80 mg treatment; e, k, diabetic + Andro 50 mg treatment; f, l, diabetic + glibenclamide 1.2 mg treatemnt. B, Statistic analysis of average area of per islets among different groups (n = 6). *P < 0.01 *vs*. normal group, **P < 0.01 *vs*. diabetic group.

The Islets of Langerhans of vehicle-treated normal mice are large and oval-shaped (Fig. 3a). In sharp contrast, in diabetic mice, the beta cell mass was obviously reduced (Fig. 3b). At both the 20 and 80 mg/kg dose levels, AL-1 demonstrated significant protection of the beta cell mass (Fig. 3c, d), and the effect was dose-dependent. The parent compound Andro and the positive control glibenclamide were also protective (Fig. 3e, f). These results suggest that the hypoglycemic effects afforded by AL-1 is at least in part due to its ability to protect the beta cell mass.

Immunohistochemical staining using an anti-insulin antibody demonstrates substantial staining in the healthy islets of Langerhans in the pancreata of normal mice compared to the much-reduced staining in the insulinopenic diabetic animals (Fig. 3g–l). Experimental diabetic animals demonstrated insulin staining in the following order: non-diabetic normals > diabetic + AL-1 80 mg/kg > diabetic + Andro 50 mg/kg > diabetic + AL-1 20 mg/kg > untreated diabetic. These results demonstrated beta cell insulin was maintained among diabetic animals treated with AL-1 and Andro. Surprisingly, although glibenclamide was shown to protect beta cell mass (Fig. 3f), only low levels of insulin staining was found in the diabetic animals receiving glibenclamide (Fig. 3l).

### AL-1 stimulates GLUT4 translocation in the plasma membrane

**Figure 4:**
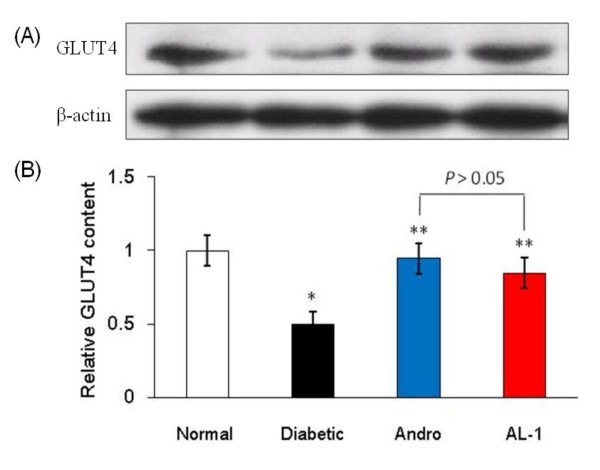
**AL-1 elevated GLUT4 translocation to the plasma membrane of soleus muscles**. Alloxan-induced diabetic mice were treated with AL-1 at 80 mg/kg, Andro at 50 mg/kg or vehicle control by intragastric administration once daily for 6 days. The soleus muscles were isolated and GLUT4 contents in plasma membrane were analyzed by western blot. (A) shows representative GLUT4 protein bands at 54 kDa; (B) shows the relative GLUT4 content normalized by internal standard, β-actin. *P < 0.05 *vs*. normal group, **P < 0.05 *vs*. diabetic group, n = 6.

Glucose transport, which depends on insulin-stimulated translocation of glucose carriers within the cell membrane, is the rate-limiting step in carbohydrate metabolism of skeletal muscle [19]. Glucose transporters mediate glucose transport across the cell membrane. GLUT4 is the predominant form in skeletal muscle [20]. Diabetes is characterized by reduced insulin-mediated glucose uptake associated with reduced GLUT4 expression [21]. In diabetic models, Andro and LA are both known to reduce blood glucose levels via upregulation of GLUT4 expression [11,22]. In the present study, the effect of AL-1 on GLUT4 content in the plasma membrane of isolated soleus muscles of diabetic mice was measured by western blot analysis. The protein level of GLUT4 in the soleus muscles of diabetic mice was only 49.5% that of the non-diabetic mice (Fig. 4; *p *< 0.05 compared with normal controls). Treatment of the diabetic mice with Andro (50 mg/kg) or AL-1 (80 mg/kg) for 6 days elevated GLUT4 protein levels to 94.6% and 84.7%, respectively, of that of the non-diabetic mice (Fig. 4; *p *< 0.05 compared with diabetic control). There was no significant difference between AL-1 and Andro treated group.

### AL-1 prevents H_2_O_2_-induced RIN-m cell death

**Figure 5:**
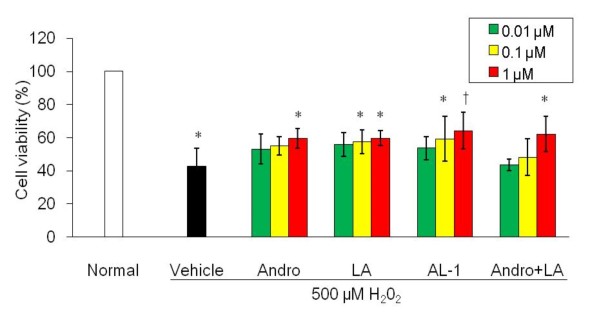
**Effect of AL-1 on H_2_O_2_-induce RIN-m cell viability**. RIN-m cells were pretreated with Andro, LA, AL-1 or Andro + LA (0.01–1 μM) following stimulation with 500 μM H_2_O_2_for 24 h. Then cell viability was determined by MTT assay. Results were expressed as the % of optical density of normal group (non-H_2_O_2 _+ vehicle treated), n = 8 replicates per group. *P < 0.01 *vs*. non-H_2_O_2 _treated group, **P < 0.05 and † *P *< 0.01 *vs*. H_2_O_2 _treated group.

Alloxan produces ROS which contribute to destruction of pancreatic beta cells, leading to diabetes. The ability of AL-1 to protect RIN-m pancreatic cells from H_2_O_2_-induced oxidative damage was studied. The viability of RIN-m cells cultured 24 h with 500 μM H_2_O_2 _was reduced to 42.7 ± 11.1% (Fig. 5). Pretreatment of the H_2_O_2_-treated RIN-m cells with Andro, LA, AL-1 or a mixture of Andro and LA at 0.01, 0.1 and 1 μM 30 min prior to H_2_O_2 _exposure for 60 min, provided significant protection. The viabilities of cells at 24 h when incubated with 1 μM concentrations of Andro, LA, AL-1 or a mixture of Andro and LA was 59.7 ± 5.9%, 59.7 ± 4.4%, 64.3 ± 11% and 62.2 ± 10.6% respectively. AL-1 was more effective than either Andro or LA. At 0.1 μM, only LA and AL-1 provided a significant protective effect. The protective effect of AL-1 was concentration-dependent. The effect of the mixture of Andro and LA was not better than AL-1, demonstrating that AL-1 was more than a simple mixture of Andro and LA.

### AL-1 quenches ROS production induced by high glucose and glibenclamide

**Figure 6:**
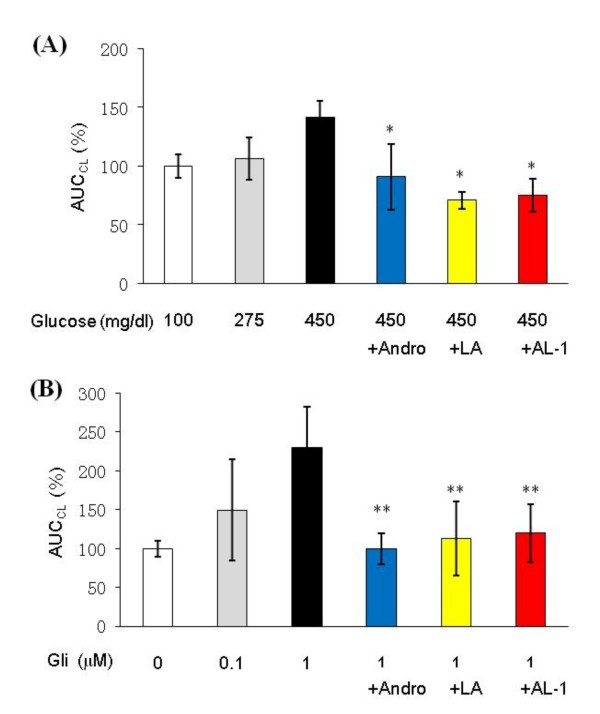
**AL-1 effectively quenched ROS production induced by high glucose and glibenclamide**. RIN-m cells were pretreated with Andro, LA or AL-1 (1 μM) following stimulation with high glucose (275 and 450 mg/dl) or glibenclamide (0.1 and 1 μM) for 2 h. Then the ROS production was measured. Results were calculated by % of AUC_CL _at 100 mg/ml glucose and 0 μM glibenclamide. (A) ROS production induced by high glucose. *P < 0.05 *vs*. 450 mg/dl glucose treatment alone; (B) ROS production induced by glibenclamide (Gli). **P < 0.05 *vs*. 1 μM glibenclamide treatment alone, n = 8 replicates per group.

High concentrations of glucose stimulate ROS production both *in vitro *[23] and *in vivo *[24,25]. ROS subsequently impair cellular function and activate apoptosis signaling, leading to beta cell damage and death [26]. To investigate the effect of AL-1 on glucose-induced ROS production *in vitro*, RIN-m cells were incubated in the presence of high concentrations of glucose, and the production of ROS was measured. Exposure of RIN-m cells to increasing concentrations of glucose (100–450 mg/dl) for 2 h increased ROS production in a concentration-dependent manner. Pretreatment of the cells with 1 μM of Andro, LA or AL-1 effectively quenched the production of increased ROS. AL-1 and LA were equally effective but more potent than Andro (Fig. 6a).

Glibenclamide treatment decreases hyperglycemia in alloxan-induced diabetic animals (Tab. 1) and protects beta cell mass from significant loss (Fig. 3f). However, the pancreatic beta cells of the glibenclamide-treated diabetic have reduced immunoreactive insulin (Fig. 3l). To understand these results, RIN-m cells were incubated with glibenclamide at increasing concentrations, and ROS production was measured. Glibenclamide dose-dependently increased ROS production (Fig. 6b), a finding previously reported [27]. Iwakura *et al*.[28] reported that viability of RIN-m cells was decreased in a dose-dependent manner by continuous exposure to glibenclamide at concentrations of 0.1 to 100 μM. When the cells were incubated in the presence of both 1 μM glibenclamide and 1 μM of Andro, LA or AL-1, the ROS induced by glibenclamide were almost completely eliminated (Fig. 6b).

### AL-1 inhibits NF-κB activation induced by IL-1β and IFN-γ inRIN-m cells

**Figure 7:**
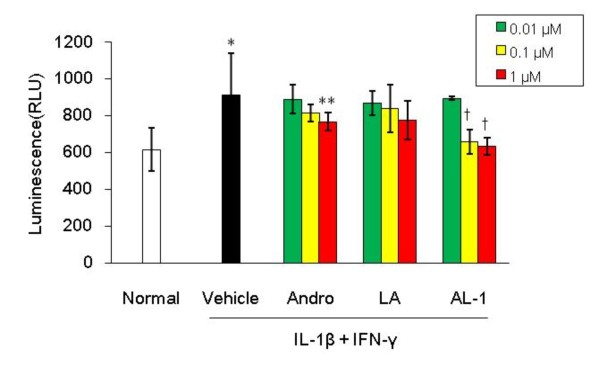
**AL-1 inhibited NF-κB activation stimulated by IL-1β and IFN-γ in RIN-m cells**. RIN-m cells were co-transfected by pNF-κB-luc and PRL-TK plasmids. After pretreament with 0.01–1 μM Andro, LA or AL-1, cells then were stimulated by IL-1β (5 ng/ml) and IFN-γ (50 ng/ml) for 24 h. NF-κB activity was detected by DLR kit. *P < 0.01 *vs*. normal control, **P < 0.05 and † *P *< 0.01 *vs*. vehicle control, n = 8 replicates per group.

Activation of NF-κB impairs the function of beta cells and contributes to cellular death [29,30]. A NF-κB reporter assay was used to investigate the effect of AL-1 on NF-κB activation. Cells were co-transfected with pNF-κB-luc and PRL-TK plasmids, pre-incubated with Andro, LA, AL-1 or vehicle followed by addition of IL-1β and IFN-γ. AL-1 at 0.1 and 1 μM significantly inhibited luciferase activity of the NF-κB reporter construct (Fig. 7; *p *< 0.01 compared with vehicle control). In fact, at 1 μM, AL-1 completely blocked IL-1β and IFN-γ-induced NF-κB activation. By contrast, Andro showed substantial NF-κB inhibition only at the highest concentration of 1 μM. AL-1 was at least 10-fold more potent than the parent compound Andro in this experiment.

Hidalgo et al. [31] reported that Andro at 5 and 50 μM significantly inhibited PAF-induced luciferase activity in a NF-κB reporter construct. Zhang and Frei [32] found that preincubation of human aortic endothelial cells for 48 h with LA (0.05–1 mM) inhibited TNF-α (10 U/ml)-induced NF-κB binding activity in a dose-dependent manner. In the presence of 0.5 mM LA, the TNF-α-induced NF-κB activation was inhibited by 81%. Thus, in the present experiment, a 1 μM concentration of LA may be too low to suppress NF-κB activation.

## Discussion

AL-1 is a new chemical entity derived by covalently linking andrographolide and lipoic acid, two molecules previously shown to have anti-diabetic properties [7,11,13-15]. In the present study, we demonstrate that alloxan-induced diabetic mice treated with AL-1 have 1) normalized blood glucose levels; 2) augmented blood insulin levels; 3) protected beta cell mass and function. These data suggest that AL-1 is a potential new anti-diabetic agent.

Types 1 diabetes is characterized by the loss of pancreatic beta cells. A novel anti-diabetic agent must have a strong hypoglycemic effect; however, the optimal agent must also be able to protect and preserve pancreatic beta cell mass and function. In our experiments, alloxan was used to induce diabetes. Alloxan produces oxygen free radicals to induce dysfunction and death of pancreatic beta cells [33]. It is known that alloxan-induced hyperglycemia can be reversible due to regeneration of beta cells, and the regeneration is early, i.e., in days [34,35]. Based on these findings, we thought that when the animals were administered alloxan, their pancreatic beta cells were damaged but the limiting threshold for reversibility of decreased beta cell mass had not been passed. AL-1, given 3 days after alloxan administration, quickly lowered blood glucose, leading to a reduction of the damaging ROS and thereby protecting beta cells from further damage and facilitated their regeneration. For the same reasons,Andro and glibenclamide also stimulated beta cell regeneration.

When an anti-insulin antibody was applied to the beta cells, we found that the beta cells of the AL-1 treated animals have significant amounts of insulin, suggesting that these cells can secrete insulin. In a sharp contrast to the AL-1-treated animals, we found little insulin in the pancreata of the glibenclamide-treated animals despite the fact that these animals had fairly large beta cell mass (Fig. 3), suggesting that the ability of these beta cells to secrete insulin has been impaired. However, results as depicted in Fig. 2 showed that the glibenclamide-treated animals had insulin levels comparable to those of the AL-1 treated animals. The reason behind the discrepancy between these results is not known at the present time, and needs to be further investigated.

Antioxidants such as N-acetyl-L-cysteine, vitamin C, vitamin E, and various combinations of these agents have been known to protect islet beta cells in diabetic animal models [36]. Previous studies have shown that Andro and LA are both potent antioxidants [37,38]. Results in Fig. 5 show that AL-1 had protective effects toward H_2_O_2_-induced oxidative damage in RIN-m cells at concentrations from 0.01–1 μM, which are achievable in animals. Thus, it is likely that, in diabetic animals, AL-1 functions as an antioxidant to quench ROS and protect beta cells. This point is further supported by data in Fig. 6a, where AL-1 markedly suppressed glucose-induced ROS production in RIN-m cells at 1 μM. In contrast to what is found with AL-1, glibenclamide stimulated ROS production at a low concentration of 0.1 μM (Fig. 6b). AL-1, Andro or LA at 1 μM completely quenched the ROS induced by 1 μM of glibenclamide. These data and those reported by others [27,28] provide a likely explanation to the notion that there were a significant amount of insulin in the AL-1 treated mice but not in those treated with glibenclamide.

Previous investigations suggest that increased oxidative stress and NF-κB activation are potential mechanisms of action for hyperglycemic toxicity on pancreatic beta cells (([39,40]. *In vitro *evidence suggests that activation of NF-κB contributes to triggering of beta cell apoptosis [29]. The fact that AL-1 completely suppressed IL-1β and IFN-γ stimulated NF-κB expression at concentrations ranging from 0.1 to 1 μM (Fig. 7) and that overexpression of NF-κB leads to overproduction of ROS [41,42] suggest that AL-1 reduces ROS production by inhibiting NF-κB activation in addition to directly scavenging ROS through its anti-oxidative properties.

Andro is reported to react with the SH group of cysteine 62 on the p50 subunit of the NF-κB, which blocks the binding of NF-κB to the promoters of their target genes, preventing NF-κB activation [43]. LA was reported to inhibit NF-κB activation via modulation of the cellular thioredoxin system [44] or by direct interaction with the target DNA [45]. Further studies are needed to uncover how the combination drug AL-1 inhibits NF-κB.

Both Andro [11,46] and LA [22] are reported to lower blood glucose levels of diabetic animals by increasing GLUT4 expression. Western blot analysis of soleus muscle confirmed that both Andro and AL-1 treatment resulted in significantly elevated levels of GLUT4 protein. These data suggest that AL-1 stimulated GLUT4 translocation in the plasma membrane of soleus muscles, leading to increased glucose utilization. Andro has been reported to lower blood glucose via the alpha-adrenoceptor [46] or by inhibition of alpha-glycosidase [47]. In present studies, Andro at 50 mg/kg lowered blood glucose and stimulated GLUT4 translocation. Because the reported IC_50 _for Andro-inhibition of alpha-glycosidase is above 100 μM, this is unlikely to be the mechanism; however, further mechanistic studies are indicated.

## Conclusion

The actions of AL-1 can be summarized as follows: to lower blood glucose, AL-1 protects beta cell mass and preserves their insulin-secreting function, and stimulates GLUT4 translocation to increase glucose utilization. For beta cell protection, AL-1 directly scavenges ROS through its antioxidant properties and reduces ROS production by inhibiting activation of NF-κB. Although most clinically useful anti-diabetic agents reduce blood glucose levels directly or indirectly, few are reported to also protect and preserve beta cell mass and insulin-secreting functions. AL-1 possesses both of these capabilities via multiple mechanisms. Further studies to explore the mechanisms of action of this promising new anti-diabetic agent are warranted.

## Abbreviations

*A. paniculata*: *Andrographis paniculata*; Andro: andrographolide; AL-1: andrographolide-lipoic acid conjugate; DAB: 3, 3'-diaminobenzidine; DLR: dual luciferase reporter; DMSO: dimethyl sulfoxide; GLUT4: glucose transporter subtype 4; HRP: horseradish peroxidase; IFN-γ: interferon gamma; IL-1β: interleukin-1beta; LA: alpha-lipoic acid; NF-κB: nuclear factor kappa B; PMSF: phenylmethylsulfonyl fluoride; ROS: reactive oxidative species; STZ: streptozotocin.

## Competing interests

This work was partially supported by grants from the Natural Science Fund of China (30772642 to Y. W) and the Science and Technology Plans of Guangzhou City (2006Z3-E4071 to Y. W). Otherwise the authors have no competing interests.

## Authors' contributions

YW and JJ conceived the study and YW and PY designed the cellular and animal experiments. ZZ and XZ carried out the cell culture experiments and *in vivo *animal experiments. ZZ and YW drafted the final version of the manuscript. JL revised the manuscript and added critical content to the discussion. All authors have read and approved the final manuscript.

## References

[B1] Rother KI Diabetes treatment – bridging the divide N Engl J Med 2007 356 1499 1501 17429082 10.1056/NEJMp078030 PMC4152979

[B2] Porte D Jr Banting lecture 1990. Beta-cells in type II diabetes mellitus Diabetes 1991 40 166 180 1991568 10.2337/diab.40.2.166

[B3] Hao E Tyrberg B Itkin-Ansari P Lakey JR Geron I Monosov EZ Barcova M Mercola M Levine F Beta-cell differentiation from nonendocrine epithelial cells of the adult human pancreas Nat Med 2006 12 310 316 16491084 10.1038/nm1367

[B4] Zhao HY Fang WY Antithrombotic effects of Andrographis paniculata nees in preventing myocardial infarction Chin Med J (Engl) 1991 104 770 775 1935360

[B5] Puri A Saxena R Saxena RP Saxena KC Srivastava V Tandon JS Immunostimulant agents from Andrographis paniculata J Nat Prod 1993 56 995 999 8377022 10.1021/np50097a002

[B6] Zhang CY Tan BK Hypotensive activity of aqueous extract of Andrographis paniculata in rats Clin Exp Pharmacol Physiol 1996 23 675 678 8886488 10.1111/j.1440-1681.1996.tb01756.x

[B7] Zhang XF Tan BK Antihyperglycaemic and anti-oxidant properties of Andrographis paniculata in normal and diabetic rats Clin Exp Pharmacol Physiol 2000 27 358 363 10831236 10.1046/j.1440-1681.2000.03253.x

[B8] Shen YC Chen CF Chiou WF Andrographolide prevents oxygen radical production by human neutrophils: possible mechanism(s) involved in its anti-inflammatory effect Br J Pharmacol 2002 135 399 406 11815375 10.1038/sj.bjp.0704493 PMC1573154

[B9] Borhanuddin M Shamsuzzoha M Hussain AH Hypoglycaemic effects of Andrographis paniculata Nees on non-diabetic rabbits Bangladesh Med Res Counc Bull 1994 20 24 26 7880153

[B10] Zhang XF Tan BK Anti-diabetic property of ethanolic extract of Andrographis paniculata in streptozotocin-diabetic rats Acta Pharmacol Sin 2000 21 1157 1164 11603293

[B11] Yu BC Hung CR Chen WC Cheng JT Antihyperglycemic effect of andrographolide in streptozotocin-induced diabetic rats Planta Med 2003 69 1075 1079 14750020 10.1055/s-2003-45185

[B12] Koranyi L James D Mueckler M Permutt MA Glucose transporter levels in spontaneously obese (db/db) insulin-resistant mice J Clin Invest 1990 85 962 967 2312736 10.1172/JCI114526 PMC296517

[B13] Nanduri S Pothukuchi S Rajagopal S Akella V Pillai SB Chakrabarti R Anticancer compounds: process for their preparation and pharmaceutical composition containing them United States Patent: Dr. Reddy's Research Foundation 2002 6 486

[B14] Kamenova P Improvement of insulin sensitivity in patients with type 2 diabetes mellitus after oral administration of alpha-lipoic acid Hormones (Athens) 2006 5 251 258 17178700 10.14310/horm.2002.11191

[B15] Yi X Maeda N alpha-Lipoic acid prevents the increase in atherosclerosis induced by diabetes in apolipoprotein E-deficient mice fed high-fat/low-cholesterol diet Diabetes 2006 55 2238 2244 16873686 10.2337/db06-0251

[B16] Jiang X Yu P Jiang J Zhang Z Wang Z Yang Z Tian Z Wright SC Larrick JW Wang Y Synthesis and evaluation of antibacterial activities of andrographolide analogues Eur J Med Chem 2009 44 2936 2943 19152987 10.1016/j.ejmech.2008.12.014

[B17] Takeuchi K McGowan FX Jr Glynn P Moran AM Rader CM Cao-Danh H del Nido PJ Glucose transporter upregulation improves ischemic tolerance in hypertrophied failing heart Circulation 1998 98 II234 II239 9852908

[B18] Gazdar AF Chick WL Oie HK Sims HL King DL Weir GC Lauris V Continuous, clonal, insulin- and somatostatin-secreting cell lines established from a transplantable rat islet cell tumor Proc Natl Acad Sci USA 1980 77 3519 3523 6106192 10.1073/pnas.77.6.3519 PMC349648

[B19] Ziel FH Venkatesan N Davidson MB Glucose transport is rate limiting for skeletal muscle glucose metabolism in normal and STZ-induced diabetic rats Diabetes 1988 37 885 890 3290006 10.2337/diab.37.7.885

[B20] Pessin JE Bell GI Mammalian facilitative glucose transporter family: structure and molecular regulation Annu Rev Physiol 1992 54 911 930 1562197 10.1146/annurev.ph.54.030192.004403

[B21] Berger J Biswas C Vicario PP Strout HV Saperstein R Pilch PF Decreased expression of the insulin-responsive glucose transporter in diabetes and fasting Nature 1989 340 70 72 2739728 10.1038/340070a0

[B22] Konrad D Somwar R Sweeney G Yaworsky K Hayashi M Ramlal T Klip A The antihyperglycemic drug alpha-lipoic acid stimulates glucose uptake via both GLUT4 translocation and GLUT4 activation: potential role of p38 mitogen-activated protein kinase in GLUT4 activation Diabetes 2001 50 1464 1471 11375349 10.2337/diabetes.50.6.1464

[B23] Gleason CE Gonzalez M Harmon JS Robertson RP Determinants of glucose toxicity and its reversibility in the pancreatic islet beta-cell line, HIT-T15 Am J Physiol Endocrinol Metab 2000 279 E997 1002 11052953 10.1152/ajpendo.2000.279.5.E997

[B24] Ling Z Kiekens R Mahler T Schuit FC Pipeleers-Marichal M Sener A Kloppel G Malaisse WJ Pipeleers DG Effects of chronically elevated glucose levels on the functional properties of rat pancreatic beta-cells Diabetes 1996 45 1774 1782 8922365 10.2337/diab.45.12.1774

[B25] Tang C Han P Oprescu AI Lee SC Gyulkhandanyan AV Chan GN Wheeler MB Giacca A Evidence for a role of superoxide generation in glucose-induced beta-cell dysfunction in vivo Diabetes 2007 56 2722 2731 17682092 10.2337/db07-0279

[B26] Robertson RP Chronic oxidative stress as a central mechanism for glucose toxicity in pancreatic islet beta cells in diabetes J Biol Chem 2004 279 42351 42354 15258147 10.1074/jbc.R400019200

[B27] Tsubouchi H Inoguchi T Inuo M Kakimoto M Sonta T Sonoda N Sasaki S Kobayashi K Sumimoto H Nawata H Sulfonylurea as well as elevated glucose levels stimulate reactive oxygen species production in the pancreatic beta-cell line, MIN6-a role of NAD(P)H oxidase in beta-cells Biochem Biophys Res Commun 2005 326 60 65 15567152 10.1016/j.bbrc.2004.10.201

[B28] Iwakura T Fujimoto S Kagimoto S Inada A Kubota A Someya Y Ihara Y Yamada Y Seino Y Sustained enhancement of Ca(2+) influx by glibenclamide induces apoptosis in RINm5F cells Biochem Biophys Res Commun 2000 271 422 428 10799313 10.1006/bbrc.2000.2616

[B29] Eldor R Yeffet A Baum K Doviner V Amar D Ben-Neriah Y Christofori G Peled A Carel JC Boitard C Conditional and specific NF-kappaB blockade protects pancreatic beta cells from diabetogenic agents Proc Natl Acad Sci USA 2006 103 5072 5077 16551748 10.1073/pnas.0508166103 PMC1458796

[B30] Zeender E Maedler K Bosco D Berney T Donath MY Halban PA Pioglitazone and sodium salicylate protect human beta-cells against apoptosis and impaired function induced by glucose and interleukin-1beta J Clin Endocrinol Metab 2004 89 5059 5066 15472206 10.1210/jc.2004-0446

[B31] Hidalgo MA Romero A Figueroa J Cortes P Concha II Hancke JL Burgos RA Andrographolide interferes with binding of nuclear factor-kappaB to DNA in HL-60-derived neutrophilic cells Br J Pharmacol 2005 144 680 686 15678086 10.1038/sj.bjp.0706105 PMC1576048

[B32] Zhang WJ Frei B Alpha-lipoic acid inhibits TNF-alpha-induced NF-kappaB activation and adhesion molecule expression in human aortic endothelial cells Faseb J 2001 15 2423 2432 11689467 10.1096/fj.01-0260com

[B33] Szkudelski T The mechanism of alloxan and streptozotocin action in B cells of the rat pancreas Physiol Res 2001 50 537 546 11829314

[B34] Chakravarthy BK Gupta S Gode KD Functional beta cell regeneration in the islets of pancreas in alloxan induced diabetic rats by (-)-epicatechin Life Sci 1982 31 2693 2697 6759833 10.1016/0024-3205(82)90713-5

[B35] Rooman I Bouwens L Combined gastrin and epidermal growth factor treatment induces islet regeneration and restores normoglycaemia in C57Bl6/J mice treated with alloxan Diabetologia 2004 47 259 265 14666367 10.1007/s00125-003-1287-1

[B36] Kaneto H Kajimoto Y Miyagawa J Matsuoka T Fujitani Y Umayahara Y Hanafusa T Matsuzawa Y Yamasaki Y Hori M Beneficial effects of antioxidants in diabetes: possible protection of pancreatic beta-cells against glucose toxicity Diabetes 1999 48 2398 2406 10580429 10.2337/diabetes.48.12.2398

[B37] Shen YC Chen CF Chiou WF Suppression of rat neutrophil reactive oxygen species production and adhesion by the diterpenoid lactone andrographolide Planta Med 2000 66 314 317 10865445 10.1055/s-2000-8537

[B38] Packer L Witt EH Tritschler HJ alpha-Lipoic acid as a biological antioxidant Free Radic Biol Med 1995 19 227 250 7649494 10.1016/0891-5849(95)00017-r

[B39] Ho E Bray TM Antioxidants, NFkappaB activation, and diabetogenesis Proc Soc Exp Biol Med 1999 222 205 213 10601879 10.1046/j.1525-1373.1999.d01-137.x

[B40] Ho E Quan N Tsai YH Lai W Bray TM Dietary zinc supplementation inhibits NFkappaB activation and protects against chemically induced diabetes in CD1 mice Exp Biol Med (Maywood) 2001 226 103 111 11446433 10.1177/153537020122600207

[B41] Kwon KB Kim EK Jeong ES Lee YH Lee YR Park JW Ryu DG Park BH Cortex cinnamomi extract prevents streptozotocin- and cytokine-induced beta-cell damage by inhibiting NF-kappaB World J Gastroenterol 2006 12 4331 4337 16865774 10.3748/wjg.v12.i27.4331 PMC4087743

[B42] Xia YF Ye BQ Li YD Wang JG He XJ Lin X Yao X Ma D Slungaard A Hebbel RP Andrographolide attenuates inflammation by inhibition of NF-kappa B activation through covalent modification of reduced cysteine 62 of p50 J Immunol 2004 173 4207 4217 15356172 10.4049/jimmunol.173.6.4207

[B43] Wei Y Sowers JR Clark SE Li W Ferrario CM Stump CS Angiotensin II-induced skeletal muscle insulin resistance mediated by NF-kappaB activation via NADPH oxidase Am J Physiol Endocrinol Metab 2008 294 E345 351 18073321 10.1152/ajpendo.00456.2007

[B44] Sen CK Cellular thiols and redox-regulated signal transduction Curr Top Cell Regul 2000 36 1 30 10842745 10.1016/s0070-2137(01)80001-7

[B45] Lee HA Hughes DA Alpha-lipoic acid modulates NF-kappaB activity in human monocytic cells by direct interaction with DNA Exp Gerontol 2002 37 401 410 11772527 10.1016/s0531-5565(01)00207-8

[B46] Yu BC Chang CK Su CF Cheng JT Mediation of beta-endorphin in andrographolide-induced plasma glucose-lowering action in type I diabetes-like animals Naunyn Schmiedebergs Arch Pharmacol 2008 377 529 540 18080810 10.1007/s00210-007-0240-0

[B47] Xu HW Dai GF Liu GZ Wang JF Liu HM Synthesis of andrographolide derivatives: a new family of alpha-glucosidase inhibitors Bioorg Med Chem 2007 15 4247 4255 17428667 10.1016/j.bmc.2007.03.063

